# Multi-Institutional Analysis of Synchronous Prostate and Rectosigmoid Cancers

**DOI:** 10.3389/fonc.2020.00345

**Published:** 2020-03-24

**Authors:** Corbin D. Jacobs, Jacob Trotter, Manisha Palta, Michael J. Moravan, Yuan Wu, Christopher G. Willett, W. Robert Lee, Brian G. Czito

**Affiliations:** ^1^Department of Radiation Oncology, Duke University Medical Center, Durham, NC, United States; ^2^Department of Radiation Oncology, Durham Veteran Affairs Medical Center, Durham, NC, United States; ^3^Department of Biostatistics and Bioinformatics, Duke University Medical Center, Durham, NC, United States

**Keywords:** synchronous, prostate cancer, rectal cancer, radiation therapy, anastomotic leak

## Abstract

**Purpose:** To perform a multi-institutional analysis of patients with synchronous prostate and rectosigmoid cancers.

**Materials and Methods:** A retrospective review of Duke University and Durham Veterans Affairs Medical Center records was performed for men with both prostate and rectosigmoid adenocarcinomas from 1988 to 2017. Synchronous presentation was defined as symptoms, diagnosis, or treatment of both cancers within 12 months of each other. The primary study endpoint was overall survival. Univariate and multivariable Cox regression was performed.

**Results:** Among 31,883 men with prostate cancer, 330 (1%) also had rectosigmoid cancer and 54 (16%) of these were synchronous. Prostate cancer was more commonly the initial diagnosis (59%). Fifteen (28%) underwent prostatectomy or radiotherapy before an established diagnosis of rectosigmoid cancer. Stage I, II–III, or IV rectosigmoid cancer was present in 26, 57, and 17% of men, respectively. At a median follow-up of 43 months, there were 18 deaths due rectosigmoid cancer and two deaths due to prostate cancer. Crude late grade ≥3 toxicities include nine (17%) gastrointestinal and six (11%) genitourinary. Two anastomotic leaks following low anterior resection occurred in men who received a neoadjuvant radiotherapy prostate dose of 70.6–76.4 Gy. Rectosigmoid cancer stages II–III (HR 4.3, *p* = 0.02) and IV (HR 16, *p* < 0.01) as well as stage IV prostate cancer (HR 31, *p* < 0.01) were associated with overall survival on multivariable analysis.

**Conclusions:** Synchronous rectosigmoid cancer is a greater contributor to mortality than prostate cancer. Men aged ≥45 with localized prostate cancer should undergo colorectal cancer screening prior to treatment to evaluate for synchronous rectosigmoid cancer.

## Introduction

Colorectal cancer (CRC) and prostate cancer (PC) are among the most common malignancies worldwide, representing two of the top three most frequently diagnosed malignancies for men (1) Because of their high frequency, it is not uncommon for men to be diagnosed with both malignancies during their lifetime (2). Approximately one-sixth of men ≥50 years old with >10 years life expectancy undergoing PC screening prior to rectal cancer resection were noted to have a synchronous prostatic malignancy (3). Conversely, in men with newly diagnosed PC, screening colonoscopies identified synchronous CRC in >3% of men in one study (4).

Synchronous presentation of both malignancies is less common than metachronous presentation; however, synchronous rates are increasing. Reasons for this increase are multifactorial, including increased screening for both malignancies, improved life expectancy, increased use of pelvic magnetic resonance imaging, and increased awareness (5–7). Along these lines, the American Cancer Society recently updated CRC screening guidelines for individuals at average risk to initiate regular screening at age 45 (8).

Management of synchronous PC and rectosigmoid cancer (RSC) is challenging given anatomic proximity of both malignancies and overlapping toxicity risks to surrounding tissues. There is a clinically unmet need in determining the preferred treatment for synchronous PC/RSC. A recent review article identified only 23 total published cases, with the largest case series comprised of only 10 patients (6, 9). Given the lack of prospective data to guide management, a retrospective, multi-institutional analysis was performed to analyze treatment patterns and outcomes for synchronous PC/RSC.

## Methods

### Patient Selection

Men evaluated at Duke University Medical Center and the Durham Veterans Affairs Medical Center between 1988 and 2017 with diagnoses of both PC/RSC were identified using International Classification of Diseases (ICD)−9 and−10 codes. Electronic and paper medical records were reviewed to determine synchronous vs. metachronous presentation. Synchronous presentation was defined as objective documentation of clinical symptoms (e.g., hematochezia), laboratory data [e.g., elevated prostate specific antigen (PSA) or fecal occult blood positive], pathologic diagnosis, and/or primary treatment of one malignancy occurring within 12 months of the other. Exclusion criteria included: failure to meet the synchronous presentation definition; neuroendocrine histology; and primary cancer of the cecum, ascending, transverse or descending colon as well as anal canal.

### Data Definitions

Patient age was defined as the age at the time of initial cancer diagnosis. The American Joint Committee on Cancer 7th edition TNM stage and stage group were recorded for both malignancies. For cases with sufficient information, prostate risk groups of low, favorable intermediate, unfavorable intermediate, high, and metastatic were manually assigned (10, 11). For PC patients initially opting for active surveillance prior to RSC diagnosis, the most recent PSA and staging information were documented at the time of active treatment. The highest combination of TNM-stage as well as stage group for RSC was recorded to account for downstaging effect following neoadjuvant chemoradiotherapy. Combined prostate and rectosigmoid surgeries were defined as synchronous surgery for both malignancies. Androgen deprivation therapy (ADT) was defined as surgical castration or the use of any medication lowering testosterone levels/blocking the testosterone receptor.

### Statistical Methods

The primary outcome was overall survival (OS), which was calculated from the date of latest cancer diagnosis to the date of death or last follow up. Secondary outcomes include PC biochemical failure [Phoenix definition (12)], locoregional and distant recurrence of either malignancy, cause-specific survival, and crude incidence of grade ≥3 late toxicities. RSC locoregional recurrence was defined as pelvis or regional lymph nodes recurrence following primary treatment. Distant metastases (DM) were defined as imaging evidence of non-regional nodal, osseous, or visceral metastases. Late toxicities were scored using the Common Terminology Criteria for Adverse Events, version 5.0.

Descriptive summary statistics were performed on patient, tumor and treatment-related variables. OS was estimated via the Kaplan-Meier method, and cohort comparison performed via the log-rank test (13). Univariate and multivariable Cox regression analyses were also performed. Statistical analyses were conducted using STATA 15.1 (College Station, TX), with a *p*-value ≤ 0.05 considered statistically significant.

## Results

### Patient, Tumor, and Treatment Characteristics

Among 31,883 men with PC identified, 330 (1%) also had RSC. Fifty-four (16%) of these were considered synchronous. By decade, there were 7, 16, and 31 men diagnosed with synchronous PC/RSC between 1988–1997, 1998–2007, and 2008–2017, respectively. The median age at diagnosis was 67 years. PC was more commonly the initial diagnosis (59%). Of these, 15 men underwent prostatectomy (*n* = 13) or radiotherapy (*n* = 2) prior to a diagnosis of synchronous RSC. Table 1 summarizes patient, tumor, and treatment characteristics.

**Table 1:** Patient, tumor, and treatment characteristics.

**Variable**		***N* (%) or median (IQR)**
Age (years)		67 (62–72)
Latest year of cancer diagnosis		2009 (2004–2012)
Sequence of malignant diagnoses	Prostate first	32 (59.3)
	Rectosigmoid first	18 (33.3)
	Same date	4 (7.4)
Pre-treatment prostate specific antigen (ng/mL)		10.8 (6.7–29.3)
T-stage of prostate cancer	T1b-T1c	16 (29.6)
	T2a-T2c	17 (31.5)
	T3a-T3b	9 (16.7)
	Unknown	12 (22.2)
N-stage of prostate cancer	0	46 (85.2)
	1	2 (3.7)
	Unknown	6 (11.1)
M-stage of prostate cancer	0	46 (85.2)
	1	2 (3.7)
	Unknown	6 (11.1)
Prostate cancer risk group	Low	8 (14.8)
	Favorable intermediate	8 (14.8)
	Unfavorable intermediate	10 (18.5)
	High	19 (35.2)
	Metastatic	2 (3.7)
	Unknown	7 (13.0)
T-stage of rectosigmoid cancer	T1	6 (11.1)
	T2	9 (16.7)
	T3	34 (63.0)
	T4	2 (3.7)
	Unknown	3 (5.6)
N-stage of rectosigmoid cancer	0	27 (50.0)
	1a-1b	19 (35.2)
	2a-2b	5 (9.3)
	Unknown	3 (5.6)
M-stage of rectosigmoid cancer	0	45 (83.3)
	1	9 (16.7)
Stage group of rectosigmoid cancer	1	14 (25.9)
	2-3	31 (57.4)
	4	9 (16.7)
Prostate surgical procedure	Biopsy only	33 (61.1)
	Prostatectomy	17 (31.5)
	Pelvic exenteration	1 (1.9)
	TURP	1 (1.9)
	Cryoablation	1 (1.9)
	Unknown	1 (1.9)
Rectosigmoid surgical procedure	Biopsy only	11 (20.4)
	Low anterior resection	24 (44.4)
	Abdominoperineal resection	10 (18.5)
	Transanal local excision	7 (13.0)
	Pelvic exenteration	1 (1.9)
	Unknown	1 (1.9)
Combined surgery for both malignancies^*^		2 (3.7)
Radiotherapy treatment	None for prostate cancer	24 (44.4)
	None for rectosigmoid	28 (51.9)
	Prostate gland/bed only	10 (18.5)
	Rectum and pelvic nodes without prostate gland/bed	5 (9.3)
	Both malignancies treated in the same course	19 (35.2)
	Both malignancies treated in separate courses^†^	1 (1.9)
Radiotherapy modality (n=35)	Brachytherapy alone	2 (5.7)
	3D conformal	16 (45.7)
	IMRT	11 (31.4)
	Unknown	6 (17.1)
Radiotherapy total dose (Gy)	Prostate	66 (60.7–72.1)
	Rectosigmoid	50.4 (50.4–54.0)
Radiotherapy total fractions	Prostate	34 (29–38)
	Rectosigmoid	28 (28–30)
5-FU based chemotherapy	None	18 (33.3)
	Neoadjuvant	5 (9.3)
	Concurrent with radiotherapy	23 (42.6)
	Adjuvant	13 (24.1)
	Palliative	3 (5.6)
	Unknown	2 (3.7)
Androgen deprivation therapy	None	30 (55.6)
	Yes	23 (42.6)
	Unknown	1 (1.9)
No treatment for prostate cancer		9 (16.7)

IQR, Interquartile range; TURP, Transurethral resection of the prostate.

* One patient underwent pelvic exenteration and another patient underwent combined abdominoperineal resection and open radical prostatectomy.

† *Prostate brachytherapy followed 17 months later by 3D conformal external beam radiotherapy to the rectum and elective nodes with opposed lateral fields only to avoid overlap of previously irradiated tissues. The patient stopped treatment after 37.8 Gy due to intractable diarrhea requiring hospitalization*.

Approximately half (48%) of men had low or intermediate risk PC with a median pretreatment PSA of 10.8. Two men had nodal and DM from PC at diagnosis, and both had non-metastatic RSC. Nearly one-third (32%) of men underwent prostatectomy, 65% received radiotherapy, 43% received ADT, and 17% received no PC treatment.

The majority of RSC (93%) originated in the rectum. The stage group distributions were 26, 57, and 17% stage I, II–III, and IV, respectively. Low anterior resection (LAR) was more commonly performed compared to abdominoperineal resection (APR): 44 vs. 19%. Two men underwent combined surgery for both malignancies: one pelvic exenteration and one combined APR and open radical prostatectomy.

Nearly two-thirds of men received 5-FU-based chemotherapy (63%) and/or radiotherapy (65%). When administered, the most common radiotherapeutic strategy was to treat both malignancies in the same course (19/35 [54%]). The median prostate gland/fossa and rectosigmoid doses were 66 Gy and 50.4 Gy, respectively.

### Survival Analysis

After a median follow up of 43 months, 34 deaths occurred in the entire cohort. Median OS for the entire cohort was 58 months (95% confidence interval 39–106 months). Median OS for stage groups I, II–III, and IV RSC was not reached, 78 months and 13 months, respectively (*p* < 0.001). Median OS for low/favorable intermediate risk, unfavorable intermediate risk, high risk, and metastatic PC was 73 months, not reached, 53 months, and 9 months, respectively (*p* < 0.001).

Table 2 summarizes the primary and secondary outcomes, including causes of death. More than half (53%) of deaths were attributed to progression of RSC. The next most likely cause of death was due to another non-prostate, non-rectosigmoid malignancy, specifically primary lung or hematologic malignancies (21%).

**Table 2:** Clinical events of synchronous prostate and rectosigmoid cancers.

**Variable**		***N* (%) or median (IQR)**
Follow up (months)		43 (21–93)
Prostate cancer outcomes (*n* = 54)	Biochemical failure	12 (22.2)
	Castrate resistance	3 (5.6)
	Distant metastasis	4 (7.4)
Rectosigmoid cancer outcomes (*n* = 54)	Permanent colostomy	19 (35.1)
	Locoregional recurrence	4 (7.4)
	Distant metastasis	20 (37.0)
Cause of death (*n* = 34)	Grade 5 toxicity^*^	3 (8.8)
	Prostate cancer progression	2 (5.9)
	Rectosigmoid cancer progression	18 (52.9)
	Other malignancy^†^	7 (20.6)
	Unknown cause of death without recurrence of either cancer	4 (11.8)

IQR, Interquartile range.

* One patient died from acute coronary syndrome after starting androgen deprivation therapy. Refer to Table 3 for details of grade 5 gastrointestinal and genitourinary toxicities.

† *3 deaths due to non-small cell lung cancer, 2 due to multiple myeloma, 1 due to acute myeloid leukemia, and 1 due to chronic lymphocytic leukemia and myelofibrosis*.

Three deaths were potentially attributable to treatment. One grade 5 gastrointestinal (GI) toxicity resulted from complications of hepatectomy for liver metastases. One grade 5 genitourinary (GU) toxicity occurred following combined APR and open radical prostatectomy who developed a vesicocutaneous fistula and later died from urosepsis. One patient died from acute coronary syndrome after starting ADT for metastatic PC.

Table 3 summarizes multivariable analysis results evaluating the prognostic significance of known RSC stage and PC risk groups relative to OS. Stages II–III RSC were significantly associated with decreased OS, whereas none of the risk groups of non-metastatic PC were associated with OS. Metastatic disease from either malignancy was significantly associated with decreased OS. Similar findings were noted in multivariable Cox models evaluating the prognostic significance of RSC stage and PC risk groups relative to cause-specific survival (Supplemental Table 1). Neither age at diagnosis nor year of diagnosis were prognostic for OS on univariate analysis.

**Table 3:** Univariate and multivariable Cox models relative to overall survival.

**Variable**	**Univariate (*****n*** **=** **54)**		Multivariable^*^ **(*****n*** **=** **47)**	
	**HR (95% CI)**	***p*-value**	**HR (95% CI)**	***p*-value**
**Prostate Cancer Risk Groups**				
Low/favorable intermediate risk	Reference	–	Reference	–
Unfavorable intermediate risk	0.70 (0.19–2.64)	0.599	0.94 (0.24–3.67)	0.928
High risk	1.42 (0.62–3.26)	0.410	0.75 (0.31–1.86)	0.486
Metastatic	10.99 (2.05–58.97)	0.005	31.1 (4.64–208)	<0.001
**Rectosigmoid Cancer Stage Groups**				
Stage I	Reference	–	Reference	–
Stages II–III	2.65 (0.89–7.88)	0.080	4.26 (1.22–14.9)	0.023
Stage IV	7.96 (2.20–28.88)	0.002	15.6 (3.19–76.8)	0.001
Age at diagnosis	1.03 (0.98–1.07)	0.246	–	–
Year of diagnosis	1.00 (0.94–1.06)	0.953	–	–

CI, confidence interval; HR, hazard ratio.

* *The multivariable model excluded patients with unknown prostate cancer risk group*.

### Locoregional and Biochemical Recurrence

Twelve men (22%) developed biochemical recurrence of PC and three (6%) met criteria for castrate resistance. Four men (7%) had locoregional recurrence of RSC.

### Distant Metastasis

Twenty men developed DM from RSC compared to only four from PC. The most common sites of DM from RSC were liver then lung, with two developing peritoneal carcinomatosis and two brain metastases. All DM from PC were osseous, with or without nonregional lymph nodes.

### Toxicity Analysis

Overall, the crude incidence of late grade ≥3 GI and GU toxicity was 17 and 11%, respectively. The distribution of types and grades of toxicities stratified by surgical intervention are summarized in Table 4.

**Table 4:** Late toxicities for synchronous prostate and rectosigmoid cancers.

**Late toxicity**	***N*** **(%)**			
	**Entire cohort** **(*n* = 54)**	**Prostate surgery** **(*n* = 20)**	**GI surgery** **(*n* = 42)**	**Non-operative** **management** (***n* = 9)**
Received radiotherapy	35 (64.8)	15 (75.0)	29 (69.0)	5 (55.6)
GI grade 1–2	7 (13.0)	4 (20.0)	7 (16.7)	1 (11.1)
GI grade 3–4	8 (14.8)	2 (10.0)	5 (11.9)	2 (22.2)
GI grade 5	1^*^ (1.9)	0 (0.0)	1^*^ (2.4)	0 (0.0)
GU grade 1–2	20 (37.0)	10 (50.0)	16 (38.1)	2 (22.2)
GU grade 3–4	5 (9.3)	2 (10.0)	4 (9.5)	0 (0.0)
GU grade 5	1^†^ (1.9)	1^†^ (5.0)	1^†^ (2.4)	0 (0.0)
Alpha blocker use	7 (13.0)	1 (5.0)	6 (14.3)	1 (11.1)
Erectile dysfunction medication use	6 (11.1)	5 (25.0)	6 (14.3)	0 (0.0)
Penile pump implant	2 (3.7)	2 (10.0)	2 (4.8)	0 (0.0)
Permanent colostomy	19 (35.2)	7 (35.0)	16 (38.1)	2 (22.2)
Fistula	4 (7.4)	2 (10.0)	2 (4.8)	1 (11.1)
Pelvic/femur fracture	1^°^ (1.9)	0 (0.0)	1^°^ (2.4)	0 (0.0)
Secondary malignancy	1^**^ (1.9)	0 (0.0)	1^**^ (2.4)	0 (0.0)

GI, gastrointestinal; GU, genitourinary.

* Died from complications of hepatectomy for liver metastases.

† Died from urosepsis as complication of vesicocutaneous fistula from combined abdominoperineal resection and open radical prostatectomy.

° Right intertrochanteric femur fracture following a traumatic fall 12 months after completing radiotherapy.

** *Transitional cell carcinoma of bladder diagnosed 9 years after 60 Gy of radiotherapy to the prostate and rectum*.

Approximately one-third of men underwent permanent colostomy placement: 19% due to APR and 17% due to toxicity or tumor recurrence. Four fistulas developed as a result of treatment and/or local tumor progression. As previously described, a vesicocutaneous fistula resulted from a combined APR/open prostatectomy. One iatrogenic rectourethral fistula resulted from a rectal biopsy to rule out recurrence 20 months following prostate gland intensity-modulated radiotherapy (76 Gy). Lastly, two men developed rectourethral fistulas following pelvic irradiation without colorectal surgery due to local progression of their RSC.

Eight men treated with LAR completed radiotherapy to both malignancies: six preoperatively and two postoperatively. Two treated postoperatively received a prostate boost totaling 60.4 Gy and 61 Gy, respectively, with no grade ≥3 toxicity. Two treated preoperatively with prostate external beam radiotherapy (EBRT) boost developed anastomotic leaks requiring ileostomy/colostomy: one who received 70.2 Gy and developed such within the perioperative period and another who received 76.4 Gy developed such 2.3 years following LAR. The EBRT technique in both cases was intensity-modulated radiotherapy. One patient with low risk PC received 160 Gy with low-dose rate interstitial I-125 brachytherapy seeds following LAR for T2N0M0 rectal adenocarcinoma with no evidence of disease recurrence or anastomotic leak 15 years later.

Of the 19 patients receiving concomitant radiotherapy for both malignancies in the same course, 17 received concurrent chemoradiotherapy and two sequential chemotherapy. Among these patients, two (11%) had late grade 3 GU toxicity and four (21%) had late grade 3 GI toxicity, including one patient who developed a fistula following treatment with 3D conformal radiotherapy to an unknown total dose. There were no grade 4 or 5 toxicities identified in this subgroup. Three patients ultimately required permanent ostomy: one due to salvage APR for local recurrence, one due to fistula formation, and one due to suspected anastomotic leak following ileostomy reversal. One patient developed right intertrochanteric femur fracture following a traumatic fall approximately one year following radiotherapy completion.

The entire study population demonstrated an increased risk of developing other malignancies, in addition to their known PC/RSC. Only one malignancy could be potentially attributed to radiotherapy in a patient diagnosed with transitional cell carcinoma of the bladder 9 years after receiving 60 Gy to the prostate and rectum. Other malignancies included a second/more proximal colon cancer, non-small cell lung cancer, multiple myeloma, leukemias, non-Hodgkin lymphoma, urothelial cancer of bladder or ureter, gastrointestinal stromal tumor, small bowel carcinoid tumor, larynx cancer, melanoma, and non-melanomatous skin cancers.

## Discussion

To our knowledge, this analysis represents the largest study to date of men with synchronous PC/RSC. Coordinating optimal surgery and radiotherapy for anatomically proximate cancers of the prostate and rectosigmoid region in the same patient remains challenging as the least toxic treatment approach remains unknown for this increasingly common clinical conundrum.

Twenty-eight percent of men (*n* = 15) in this analysis received treatment for non-metastatic PC prior to RSC diagnosis. Synchronous diagnosis of lower gastrointestinal malignancy could have likely been made had those individuals undergone CRC screening before undergoing prostatectomy (*n* = 13) or radiotherapy (*n* = 2). Prior to prostate radiotherapy, radiation oncologists have been advised to obtain screening colonoscopy in these patients if not performed within the past 3 years, although similar recommendations have not been widespread prior to prostatectomy (4, 14). Based on results from a prior report, the number needed to screen to diagnose one synchronous asymptomatic CRC is about 31 (4). In the current study, approximately 1% of men were noted to have a metachronous or synchronous RSC, and this figure would be higher if the entire colon were considered. The majority of these 15 men had low or intermediate risk PC in the setting of 3 stage IV and 7 stage II-III rectosigmoid adenocarcinomas. It is possible that in some of these cases, the earlier synchronous diagnosis with colonoscopy could have resulted in a lower RSC stage at diagnosis and improved survival. It is also possible many of these patients received unnecessary prostate treatment with avoidable morbidity/complicating the ability to appropriately treat the synchronous RSC.

As might be anticipated, men with metastatic disease, whether due to PC or RSC, had significantly worse OS. Additionally, RSC was the predominant cause of mortality, accounting for the vast majority of DM and over half of total deaths. On multivariable analysis, RSC stage groups II-III were significantly associated with OS, whereas non-metastatic PC risk groups were not.

The median OS for stage I RSC (which was not reached and >15 years in this study) is sufficiently long enough to warrant consideration of treatment of synchronous PC, even in low/intermediate risk groups. With a median OS of 6.5 years for patients with stage II-III RSC in this contemporary cohort, an individualized informed discussion regarding risks and benefits of treatment vs. observation for synchronous PC is recommended, as higher risk PC may warrant treatment while lower risk may not. Seventeen percent of men in this analysis received no PC-specific therapy, and it can be argued that this percentage should have been higher. Some proponents advocate for a course of ADT for selected patients with locally advanced RSC and PC in lieu of radiotherapy or surgery; however, the ADT risks of sexual dysfunction, bone demineralization, hot flashes, fatigue, sarcopenia, increased adiposity, gynecomastia, and cardiovascular complications need to be strongly considered.

**Figure 1:**
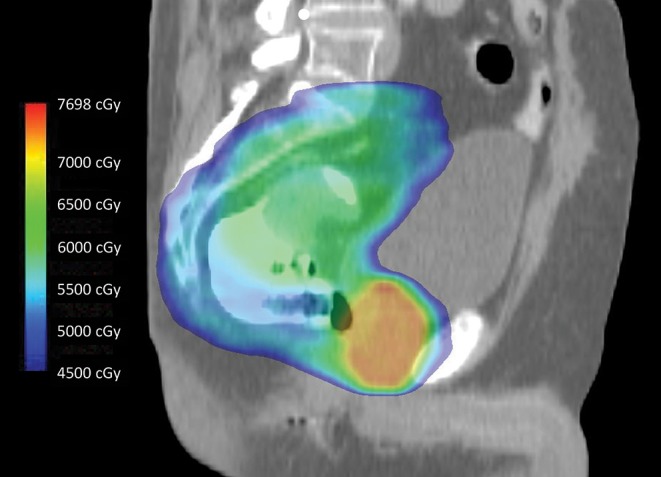
Intensity-modulated radiotherapy using simultaneous integrated boost technique to treat intact rectal and prostate cancers in 28 fractions. During treatment, the entire dose color wash volume is irradiated to differential doses on a daily basis. The primary rectal tumor, which is well visualized and surrounded by rectal contrast, receives a lower dose (green) compared to the entire prostate gland (orange-red). Note the relative sparing of the bladder anteriorly, the penile bulb inferiorly, and the small bowel superiorly.

If radiotherapy is utilized to treat RSC, a synchronous, localized PC can be treated simultaneously (Figure 1) (15). One difficulty is that PC disease-related outcomes are improved with radiation dose escalation to levels higher than what is typically given for RSC, and the associated anterior rectal wall dose can be high enough to result in proctitis as well as anastomotic leak, fistula, or stricture following RSC surgery (16, 17). Options for prostate dose escalation include interstitial brachytherapy before, during, or after rectosigmoid surgery; EBRT boost before or after surgery; or no further irradiation following standard RSC treatment. If the irradiated rectum adjacent to the prostate receiving higher boost doses is resected, as is the case with APR, then late rectal toxicity is not relevant. However, when LAR is performed in an effort to avoid permanent colostomy, late toxicity related to the high dose prostate boost, particularly the rectal segment involving the anastomosis, can result in the previously described complications and definitive stoma. Exemplifying this, two men in our analysis treated with neoadjuvant EBRT for PC/RSC with a prostate radiation boost dose to >70 Gy developed anastomotic leaks requiring surgical diversion, one of which occurred 2.3 years following LAR. Another series, which included seven men treated with 70–78 Gy preoperatively for synchronous PC followed by sphincter-preserving total mesorectal excision for rectal cancer, noted significantly increased rates of anastomotic leakage, reoperation, and definitive stoma requirement (18). In contrast, in another study with limited follow up, the only reported major complication among seven men receiving a prostate EBRT boost ≥70 Gy followed by LAR was a single case of anastomotic stricture (9). No patient in the present analysis received a prostate brachytherapy boost of any type, though a pre-LAR prostate boost with Cs-131 has been published as a proposed technique (19). Nevertheless, it is worth noting that one patient in the present analysis received definitive radiation to 160 Gy utilizing I-125 brachytherapy following LAR without complication, a dose much higher than what is conventionally administered as a boost.

A single combined operation for both malignancies, typically prostatectomy combined with either LAR or APR, can be performed (20–25). Minimally invasive surgical techniques, including laparoscopic APR (24) and robotic LAR (23), both combined with robotic-assisted prostatectomy, have been reported. In our multi-institutional analysis, two different combined open operations were performed: a pelvic exenteration as well as APR with prostatectomy. The former was successful, though potentially very morbid, whereas the second led to a vesicocutaneous fistula, repeated urinary tract infections, and eventual death from urosepsis.

Men with synchronous malignancies are at high risk of developing additional malignancies. About one-fifth (21%) of deaths in this analysis were attributable to a non-prostate/rectosigmoid, non-treatment-induced malignancy, the most common being non-small cell lung cancer and hematologic malignancies.

There are several limitations of our retrospective analysis, including incomplete medical records and selection bias. The number of patients screened for the other malignancy prior to the initial cancer diagnosis is unknown, and therefore the estimate of potential missed opportunities for earlier diagnosis is unknown. However, based on our data, about 590 men would need to be screened to identify a single man with synchronous PC and RSC. Additionally, performance status or comorbidity indices could not be included in the survival models to adjust for health status as these were not routinely documented or identifiable. Despite this being the largest study to date, the sample size remains small, particularly when limited to patients without DM at diagnosis. Furthermore, several patients had other, competing malignancies, though none of these had a documented malignancy preceding the synchronous diagnosis of PC and RSC, excluding non-melanomatous skin cancer.

## Conclusions

Men ≥45 years old with newly diagnosed non-metastatic PC should undergo CRC screening prior to treatment to evaluate for synchronous colorectal malignancy if they have not had screening performed <3 years previously. In patients with synchronous stage group II-IV RSC, the RSC is a significantly greater contributor to mortality relative to PC, and these patients may not require PC therapy. Prostate gland dose escalation with EBRT in the setting of sphincter-sparing colorectal surgery may increase the risk of anastomotic leakage and stenosis. Further investigation using interstitial brachytherapy boost techniques either pre-, intra- or post-operatively, is warranted.

## Data Availability Statement

The datasets generated for this study will not be made publicly available. This is not permitted as part of the data sharing agreement with the Veterans' Affairs. Requests to access the datasets should be directed to the corresponding author.

## Ethics Statement

The studies involving human participants were reviewed and approved by the institutional review boards at Duke University and the Durham Veterans' Affairs Medical Center. Written informed consent for participation was not required for this study in accordance with the national legislation and the institutional requirements.

## Author Contributions

CJ, WL, and BC contributed conception and design of the study. CJ, JT, and BC organized the database. CJ and YW performed the statistical analysis. CJ wrote the first draft of the manuscript. JT, MP, MM, CW, WL, and BC wrote sections of the manuscript. All authors contributed to manuscript revision, read, and approved the submitted version.

### Conflict of Interest

The authors declare that the research was conducted in the absence of any commercial or financial relationships that could be construed as a potential conflict of interest.
