# Supplementary material for: Multi-Institutional Analysis of Synchronous Prostate and Rectosigmoid Cancers
Source: Front Oncol. 2020 Mar 24;10:345. doi: 10.3389/fonc.2020.00345 (PMC7105852; doi:10.3389/fonc.2020.00345)
Supplement: Supplementary file 1 [file Table_1.docx]

**Supplementary Table 1. Multivariable Cox models relative to cause-specific survival**

| Variable | Death due to cancer | | Death not due to cancer | |
| --- | --- | --- | --- | --- |
|  | HR (95% CI) | p-value | HR (95% CI) | p-value |
| Prostate cancer risk groups  Low/favorable intermediate risk  Unfavorable intermediate risk  High risk  Metastatic | Reference  0.5 (0.1 – 4.7)  0.8 (0.3 – 2.6)  53.7 (6.1 – 476.9) | --  0.570  0.740  <0.001 | Reference  1.4 (0.2 – 8.8)  0.7 (0.1 – 2.9)  Non-convergence* | --  0.707  0.574  0.999 |
| Rectosigmoid cancer stage groups  Stage I  Stages II-III  Stage IV | Reference  5.4 (0.9 – 34.0)  26.8 (3.3 – 217.8) | --  0.072  0.002 | Reference  3.4 (0.6 – 18.8)  Non-convergence* | --  0.168  0.999 |

Abbreviations: confidence interval (CI), hazard ratio (HR)

*The estimations do not converge due to small sample size
